# Advancing wavefront sensing: meta Shack-Hartmann sensor enhances phase imaging

**DOI:** 10.1038/s41377-024-01646-4

**Published:** 2024-12-02

**Authors:** Xiaoyuan Liu, Zihan Geng, Mu Ku Chen

**Affiliations:** 1 The State Key Laboratory of Terahertz and Millimeter Waves, City University of Hong Kong, Kowloon, Hong Kong, 999077 China; 2 Department of Electrical Engineering, City University of Hong Kong, Kowloon, Hong Kong, 999077 China; 3 Institute of Data and Information, Tsinghua Shenzhen International Graduate School, Tsinghua University, Shenzhen, Guangdong 518071 China

**Keywords:** Imaging and sensing, Optical sensors

## Abstract

A meta-lens array-based Shack-Hartmann wavefront sensor has been developed to break the limits imposed by the size and curvature of traditional micro-lenses, which significantly improves both sampling density and angular resolution of phase measurement. Metasurface advances the field of optical phase measurement to smaller-scale complex wavefront characterization.

## Abstract (web summary)

A meta-lens array-based Shack-Hartmann wavefront sensor enhances phase measurement by increasing sampling density and angular resolution, advancing optical wavefront sensing with metasurface technology.

## Graphical abstract

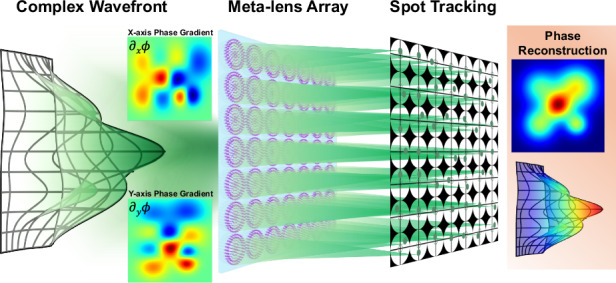

Optical phase measurements are crucial in various scientific and engineering applications as they provide detailed information about the wavefront of light, enabling precise analysis of material properties, biological structures, and optical system performance^1^. For phase map measurements, conventional phase imaging methodologies can be classified into two principal categories: interferometry-based techniques^2^ and computational phase retrieval approaches^3^. The interferometry-based phase imaging method uses interference patterns created by combining a signal beam, which illuminates the object, and a reference beam, which bypasses the object, to reconstruct a phase map with high accuracy. Computational phase retrieval methods for phase imaging reconstruct the phase information of an object by iteratively processing intensity measurements captured at different planes or under varying conditions, often relying on algorithms. In contrast to the aforementioned phase imaging methods that necessitate coherent light sources, wavefront sensing techniques offer an alternative indirect method for retrieving optical phase maps, deducing the phase map from the intensity pattern displacement^4^. The Shack-Hartmann wavefront sensor (SHWFS) represents the most commonly employed category of wavefront sensing methods^5^. SHWFS measures the phase map via the displacement of the focal spots of a lenslet array. One of the primary objectives of optical phase imaging systems is to enhance resolution, enabling the imaging of increasingly smaller features. However, the traditional micro-lens array in SHWFS has the constraints of limited minimum feature size and maximum curvature of each micro-lens, which can only characterize slowly varying wavefronts^6^. Meta-lenses, on the other hand, are engineered with nanostructures that enable precise control over light propagation at the sub-wavelength scale^7^. The flat meta-lens can be designed with specific optical properties, such as customized focal lengths and aberration corrections, which are not feasible with conventional micro-lenses. To overcome this limitation, Mooseok Jang’s group from the Korea Advanced Institute of Science and Technology (KAIST) proposed to use a meta-lens array to realize quantitative phase imaging of complex phase objects with greatly increased density and curvature of the lenslet. Beyond traditional applications like light field imaging^8^, structured light^9^, and high-dimensional quantum light sources^10^, the researchers have successfully expanded the utility of the meta-lens array in the phase imaging field.

In the recent work published in *Light: Science & Applications*^11^, Gi-Hyun Go et al. introduced a metasurface-enhanced SHWFS (meta SHWFS) based on a meta-lens array for single-shot phase imaging. The meta SHWFS achieves a sampling density of 5963 per mm^2^ and a maximum acceptance angle of 8°. The proposed meta-lens array comprises 100 × 100 meta-lenses, each with an extremely small diameter of just 12.95 µm and a relatively high NA of 0.21, representing a significant advancement over conventional micro-lens dimensions. Compared with conventional SHWFS systems, meta SHWFS achieves 100 times higher spatial resolution and 10 times greater phase gradient. The experimental error for phase imaging of complex objects is only 0.12λ.

As shown in Fig. 1, the basic principle of the meta SHWFS is the analysis of the local wavefront slopes using a meta-lens array. An image sensor is placed at the focal plane of the meta-lens array. Each meta-lens focuses a small portion of the incoming wavefront onto the sensor. In the ideal case of a perfect planar wavefront, the focal spots formed by each meta-lens would align perfectly with a predefined reference grid on the sensor. However, the phase gradients of a complex wavefront will cause different incident angles to each meta-lens. The incident angles will be reflected as the radial displacements of the focal spots. By measuring these displacements, the local wavefront gradients can be determined. Subsequently, these gradients are integrated to reconstruct the overall wavefront profile. The diameter of the meta-lens is chosen by considering the unit structure period and ensuring compatibility with the CMOS sensor to prevent undesirable diffraction effects. The determination of focal length is comprehensively based on the focal spot localization errors at various signal-to-noise levels, ensuring precise and accurate measurements. To test the performance of the proposed meta SHWFS, it was verified through two applications: three-dimensional position tracking of an LED light source to demonstrate incoherent light handling capabilities and phase imaging of complex objects to showcase its ability to characterize intricate wavefronts. These tests highlight the system’s versatility and its potential for high-resolution phase imaging in various scientific and engineering contexts.

**Fig. 1:**
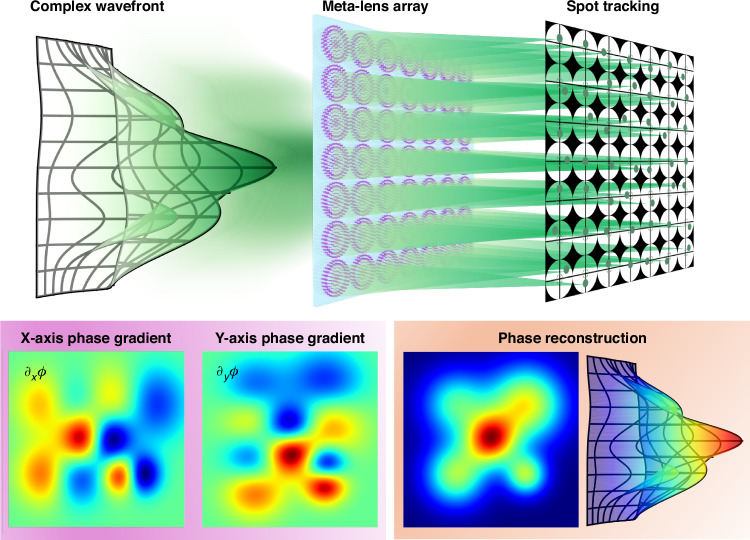
**Schematic of the wavefront sensing principle of meta-lens array-based Shack-Hartmann wavefront sensor (SHWFS)**. An incoming complex wavefront strikes the meta-lens array, with each meta-lens capturing a specific segment of the wavefront. These meta-lenses focus the incident light onto a detector, forming a pattern of focal spots. The positions of these focal spots depend on the phase gradients of the wavefront segment. By analyzing the displacement of each focal spot from its expected position, the local wavefront gradients can be computed and integrated to reconstruct the overall wavefront configuration

In summary, meta-lens array-based SHWFS displays a novel solution for higher-resolution wavefront measurements. Compared to conventional SHWFS systems using micro-lens arrays, the reduced lens diameter of the meta-lens increases the system’s sampling density, while the shorter focal length enhances both the maximum acceptance angle and the angular resolution. The showcased complex wavefront characterization capability of the proposed meta SHWFS would benefit various applications, including adaptive optics in telescopes, ophthalmology for measuring the aberrations of the human eye, and laser beam diagnostics, making it a powerful tool in both scientific research and practical applications.
